# Continuous flow insufflation of oxygen compared with manual ventilation during out-of-hospital cardiac arrest: A survey of the paramedics

**DOI:** 10.1177/20503121211018105

**Published:** 2021-06-30

**Authors:** Mathieu Groulx, Alexandra Nadeau, Marcel Émond, Jessica Harrisson, Pierre-Gilles Blanchard, Douglas Eramian, Eric Mercier

**Affiliations:** 1 Centre de recherche du CHU de Québec, Université Laval, Québec, QC, Canada; 2 Faculté de médecine, Université Laval, Québec, QC, Canada; 3 Centre de recherche sur les soins et les services de première ligne de l’Université Laval (CERSSPL-UL), Québec, QC, Canada; 4 Département de médecine d’urgence, CHU de Québec, Québec, QC, Canada; 5 Direction des services préhospitaliers d’urgence, Centre Intégré Universitaire de Santé et de Services Sociaux (CIUSSS) de la Capitale-Nationale, Québec, QC, Canada

**Keywords:** Continuous flow insufflation, cardiac arrest, airway management, paramedics, health care survey

## Abstract

**Introduction:**

In 2018, a continuous flow insufflation of oxygen (CFIO) device (b-card™, Vygon (USA)) placed on a supraglottic airway (SGA) became the standard of care to ventilate patients during adult out-of-hospital cardiac arrest (OHCA) care in Quebec–Capitale-Nationale region, Canada. This study aims to assess the paramedics’ perception as well as the disadvantages and the benefits relative to the use of CFIO during OHCA management.

**Methods:**

An invitation to complete an online survey (Survey Monkey™) was sent to all 560 paramedics who are working in our region. The survey included 22 questions of which 9 aimed to compare the traditional manual ventilation with a bag to the CFIO using a 5-point Likert-type scale.

**Results:**

A total of 244 paramedics completed the survey, of which 189 (77.5%) had used the CFIO device during an OHCA at least once. Most respondents felt that the intervention was faster (70.2%) and easier (86.5%) with the CFIO device compared with manual ventilation. CFIO was also associated with perceived increased patient safety (64.4%) as well as paramedic safety during the evacuation (88.9%) and the ambulance transport (88.9%). Paramedics reported that physical (48.1%) and cognitive (52.9%) fatigue were also improved with CFIO. The main reported barriers were the bending of the external SGA tube and the loss of capnography values.

**Conclusion:**

The use of CFIO during adult OHCA care allows a simplified approach and was perceived as safer for the patient and the paramedics compared with manual ventilation. Its impact on patient-centred outcomes needs to be assessed.

## Introduction

Out-of-hospital cardiac arrest (OHCA) remains a leading cause of death worldwide.^1^ Prompt initiation of high-quality cardiopulmonary resuscitation (CPR) and rapid access to defibrillation are critical to improve survival.^2^ The impact of ventilation during CPR on patient-important outcomes is less understood.^3^ However, ventilating a patient in accordance with the current OHCA management guidelines poses numerous challenges to health care professionals.^4^ For instance, during OHCA, ventilation often requires a dedicated paramedic. In a resource-limited environment such as the prehospital setting, this is an important issue that can lead to prolonged on-site delays. New ventilation devices that deliver a continuous flow insufflation of oxygen (CFIO) have been developed to facilitate ventilation during OHCA care. While the CFIO device maintains a positive alveolar pressure, ventilation is delivered by the concomitant chest compressions to achieve adequate gas exchanges.^5–7^ The impact of CFIO on patients’ outcomes is unclear but CFIO was associated with improved gas exchange parameters^5,7–11^ and increased coronary perfusion pressure^10^ in small human cohort studies and animal model studies.

In November 2018, in the administrative region of Quebec–Capitale-Nationale (Quebec, Canada) a CFIO device, the b-card (Vygon™),^12^ was introduced as the standard of care ventilation method during adult non-traumatic OHCA care. As per regional OHCA management protocol, the CFIO is attached to a supraglottic airway (SGA) device (Combitube™)^13^ instead of the traditional manual ventilation using a bag.^14^ It operates on the principle of creating a virtual valve generated by a gas flow. When installed, CFIO provide ventilation in conjunction with chest compressions. Therefore, no interruption of chest compressions during CPR is required. The use of CFIO to ventilate patients therefore changes the work’s dynamic of the paramedics, allowing the one paramedic to perform the resuscitation while the other can manage other priorities and prepare the evacuation to an emergency department (ED). To facilitate the implementation of new devices and change current practices, change-agents need to communicate the scientific basis for the change and underline its need and value relative to current practice to health care providers.^15^ Moreover, understanding health care professional’s response to change may be critical to the introduction of new protocols.^16–18^ Such process of evaluating paramedics’ changes of practice has been already done in the past.^19–22^ There is therefore a need to gather the paramedics’ opinion relative to the use of CFIO during CPR. This study aims to assess the paramedics’ perception relative to the use of CFIO during adult non-traumatic OHCA care and to identify the disadvantages and the benefits to its use compared with traditional manual ventilation.

## Methods

### Ethic

This study protocol was submitted to the Centre de Santé et Services Sociaux – CIUSSS de la Capitale Nationale Institutional Review Board (IRB) (2017-2018-21 MP). After reviewing the study protocol, the IRB determined that this study represented a quality assessment of an implementation project (article 2.5, Tri-Council Policy Statement: Ethical Conduct for Research Involving Humans),^23^ thus waiving the requirement for a specific individual ethical consent.

### Updated ventilation protocol

In the province of Quebec, prehospital care is delivered almost exclusively by emergency medical technicians (EMT). Prehospital treatment of OHCA includes chest compressions, defibrillation, and airway management using a SGA device, the Combitube™. Most interventions are performed by a team of two paramedics. Occasionally, a paramedic supervisor is requested to provide assistance. Paramedics are following a standardized provincial adult OHCA management protocol that was developed using the 2015 American Heart Association (AHA) guidelines.^24^ The protocol states to carry out cycle of 30 chest compressions for two ventilations or, when two paramedics are available, to complete cycle of 200 chest compressions on asynchronous ventilation following SGA device insertion. Insertion of the SGA device is performed after the second cycle. Traditionally, the ventilation was performed using a bag that requires manual compressions by a dedicated paramedic. Following the CFIO implementation, paramedics placed the CFIO device on the Combitube^TM^ following its insertion. The virtual valve of the CFIO creates an acceleration of the oxygen flow, allowing for a positive intra-thoracic pressure during the thoracic compressions. Ventilation is thus performed by the mechanical compression-decompression effect of the chest compressions. CFIO use was limited to adult following a non-traumatic OHCA situation. Prior to the implementation of the CFIO device, a mandatory 1-day training session was completed by all paramedics. The training included a 3-h didactic session on ventilation, CFIO and the new cardiac arrest management protocol, a 2-h session with small group demonstrations of the device and 2 h of simulated cardiac arrest scenarios using manikins.

During the year 2018–2019, paramedics responded to 993 OHCA in Quebec–Capitale-Nationale region and attempted resuscitation for approximately 42% of the interventions (regional data). Overall, SGA and CFIO devices are therefore used during close to half of OHCA interventions. There are approximately 560 paramedics currently working in the region of Quebec–Capitale-Nationale.

### Study design

This is a web-based survey (Survey Monkey™) study. All paramedics actively working in the Quebec–Capitale-Nationale region were invited to complete the survey on a voluntary basis. There were no exclusion criteria. The online link to the survey was shared by the Centre Intégré Universitaire de Santé et de Services Sociaux (CIUSSS) de la Capitale-Nationale. The online survey was launched 7 months following the CFIO implementation, and paramedics had 5 months to complete the survey during which five email reminders were sent. Paramedics were explicitly requested to complete the survey only once. There was no financial incentive for taking part in the survey.

### Questionnaire development process

A questionnaire was developed using a guide for the design and conduct of self-administered surveys of clinician’s knowledge, attitudes and practice.^25^ The survey was initially developed by three authors (E.M., A.N., M.G.) and later tested on five paramedics prior to distribution to determine relevance, comprehension and appropriateness of the questions. The final questionnaire included 22 questions of which nine aimed to directly compare the manual ventilation protocol with the CFIO ventilation protocol with the b-card^TM^ using a 5-point Likert-type scale (totally in agreement, partially in agreement, neutral/no difference between the two methods, partially in disagreement, totally in disagreement). An English version of the questionnaire is presented in Supplemental Appendix 1.

### Data analysis

Statistical analyses were limited to the use of central tendency and frequency measures. Comments relative to the disadvantages and the benefits were collected using the Excel™ software. As there is no survey in the literature that has been previously done regarding the use of CFIO during cardiac arrest, we used inferred categories to classify disadvantages and benefits. Narrative text relative to disadvantages and benefits were reviewed by two investigators (M.G., A.N.) who independently inferred categories. Categories inferred by the two investigators were thereafter compared. Final categorisation was made by consensus between these two authors. No specific coding strategy was used.

## Results

### Study participants

Two hundred forty-four paramedics completed the survey during the study period, of which 189 (77.5%) reported having used the CFIO device. Ninety-eight paramedics reported having used the CFIO device once while 91 reported having used the b-card^TM^ more than once. Among all paramedics currently working in the region, the overall response rate was 43.6%.

The mean age of all respondents was 33.9 years old (SD = 8.8) and 60 (24.6%) were women. A total of 228 (93.4%) paramedics consider themselves to be well trained to use or potentially use the b-card™. The characteristics of all respondents are presented in Table 1.

**Table 1.** Respondents’ characteristics (N = 244).

Age, mean (SD)	33.9 (8.8)
Female	60 (24.6)
Years of experience	
<6	81 (33.2)
6–15	117 (48.0)
>15	46 (18.8)
Number of interventions with the b-card™*	
0	54 (22.2)
1	98 (40.3)
2	52 (21.4)
3	23 (9.5)
>3	16 (6.6)

SD: standard deviation. Data are presented as N (%). *Data missing: 1.

### Comparison between the CFIO and the manual ventilation

Table 2 presents paramedics’ perceptions of CFIO for those who have used the device at least once. For those nine questions, the professionals were invited to compare the CFIO to the manual ventilation method. Most respondents felt that CFIO increased the patient safety (n = 121, 64.4%) and the paramedic safety mostly during the evacuation (n = 169, 89.9%) and the ambulance transport (n = 167, 88.8%) compared with manual ventilation. The physical (n = 91, 48.1%) and cognitive (n = 100, 52.9%) fatigue were improved, but less frequently, with CFIO. The majority of respondents felt that the airway management was faster (n = 132, 69.8%) and easier (n = 163, 86.2%) to conduct with the use of the CFIO compared with manual ventilation technique.

**Table 2.** Paramedics’ perception regarding the use of CFIO compared to manual ventilation (N = 189).

	Partially/totally in agreement	Neutral/the 2 methods are equivalent	Partially/totally in disagreement
Compared with manual ventilation, please state your level of agreement with the following statements:			
The use of CFIO is safer for the patient during transport**	121 (64.4)	50 (26.6)	16 (8.5)
The use of CFIO is safer for the paramedic during intervention**	139 (73.9)	38 (20.2)	10 (5.3)
The use of the CFIO is safer for the paramedic during transport**	167 (88.8)	11 (5.9)	9 (4.8)
The use of the CFIO is safer for the paramedic during evacuation**	169 (89.9)	11 (5.9)	7 (3.7)
The intervention is faster with the use of CFIO*	132 (69.8)	40 (21.2)	16 (8.5)
The intervention is easier with the use of CFIO*	163 (86.2)	14 (7.4)	11 (5.8)
You feel less physical fatigue with the use of CFIO*	91 (48.1)	68 (36.0)	29 (15.3)
You feel less cognitive fatigue with the use of CFIO*	100 (52.9)	60 (31.7)	28 (14.8)
You prefer the use of the CFIO*	159 (84.1)	18 (9.5)	11 (5.8)

CFIO: continuous flow insufflation of oxygen; OHCA: out-of-hospital cardiac arrest. Data are presented as N (%). **Missing data: 2. *Missing data: 1.

### Disadvantages and benefits

Using a free text narrative space, paramedics were invited to share the disadvantages and the benefits they perceived relative to the use of CFIO devices. A total of 176 respondents commented on the potential disadvantages. The main disadvantages identified were the bending of the distal end of the SGA device under the weight of the CFIO device (n = 79, 44.9%) and the loss of accurate capnography values (n = 58, 33.0%). Thirty-four of the 176 respondents (19.3%) reported having observed no disadvantages.

Among the 166 comments received relative to the potential benefits, the fact that the use of CFIO frees one operator was the most frequent benefit reported (n = 98, 59.0%). Other identified benefits were the quickest evacuation (n = 36, 21.7%), the available time to gather witness information (n = 28, 16.9%), and to complete other tasks (n = 19, 11.4%). Fourteen respondents (8.4%) reported the ease and speed with which the CFIO can be placed on the SGA device. In addition, 57 paramedics (34.3%) mentioned that CFIO device use allows them to focus on CPR quality as they do not have to count ventilations. Some paramedics reported that the ambulance transport to the hospital was safer with a CFIO device (n = 10, 6.0%), as there is no need for operator movement to ventilate inside the vehicle. Seventeen respondents (10.2%) reported that CFIO makes the intervention quicker and easier.

## Discussion

Surveyed paramedics felt that CFIO device during OHCA management improved the resuscitation in terms of safety, ease and speed when compared with the traditional manual ventilation using a bag. CFIO was also associated with a reduced feeling of physical and cognitive fatigue following the intervention. These perceived benefits are important, particularly in a limited resource environment such as the prehospital setting.

The bending of the distal end of the SGA device and the loss of accurate capnography values were the main reported disadvantage of CFIO. Concerning capnography, it has been suggested that it may be used to monitor CPR quality and predict return of spontaneous circulation (ROSC).^26,27^ Although it is not recommended to use capnography values alone for decision making in cardiac arrest management^28^ as several confounders can influence its value,^29,30^ there would be a need to evaluate whether the advantage of retrieving a paramedic for other tasks outweighs the disadvantage of no longer having this information during the intervention. An additional tool can be added to some CFIO device to obtain capnography values, but it was not readily available during the implementation in our region.

Ambulance transport is a critical moment during resuscitation and is associated with decreased CPR and ventilation quality in addition to greater risk of injuries for the paramedics. Use of CFIO could partially alleviate these problems. The difficulty of providing high-quality manual CPR while moving a patient on a stretcher or an extrication device is recognized.^31^ It can contribute to prolonged extrications. In addition, there are inherent dangers to carry out CPR while in a moving ambulance that must be considered. Important acceleration and deceleration forces can cause paramedics to lose their balance, putting them at risk of injury during work. Implementation of a mechanical chest compression system simultaneously with the use of a CFIO device could enable the paramedics to sit securely at the patient’s head while monitoring mechanical CPR and CFIO. To determine whether paramedics’ perception of the CFIO device translates into improved patient outcomes, well-designed controlled studies are required.

The impact of CFIO on patient-centred outcomes is still unclear.^5,7,11^ To our knowledge, only two trials have compared the use of CFIO to other methods of ventilation in human studies.^5,7^ These two randomized controlled trials included 95 and 696 patients and were conducted in France. They compared the impact of CFIO to manual ventilation following adult OHCA on various outcomes. In these two studies, the rate of return of spontaneous circulation (ROSC) and time to achieve ROSC were similar between CFIO and manual ventilation. Bertrand et al. reported a higher proportion of patients with measurable SpO_2_ and a higher proportion of patients with SpO_2_ values above 70%.^7^ The impact of CFIO on meaningful outcomes still needs to be rigorously assessed.

### Limitations

As several paramedics have responded to more than one case of OHCA since the implementation of the CFIO device while other were not exposed to its use during the survey period, the exact response rate is unknown (i.e. we are unable to know the exact number of paramedics who were exposed to the CFIO among the non-responders). Nevertheless, although unlikely to represent the actual response rate among those who have used the CFIO, if we calculated an overall response rate of all paramedics who work in our region, the worst response rate scenario was 43.6%. This is close to the 50% to 70% response rate reported among other health professionals.^32,33^ During the study period, it is estimated that approximately 150 b-card^TM^ were inserted. As a maximum of three paramedics are involved during an OHCA and the respondents have used the CFIO at least 335 times, the worst response rate among those who have used the CFIO is estimated to be 75% (335/450). No sample size calculation or power analysis was performed. Also, although it was explicitly requested that each paramedic could only complete the questionnaire once, it is not impossible that a paramedic have completed the survey more than once. A part of the gap in the response rate is probably due to a significant proportion of paramedics who did not perform CPR with the CFIO and therefore did not felt concerned by the survey. The variable delay between device use and the survey completion among respondents could also have an impact on their responses. Moreover, the survey was limited to EMT and respondents were mostly young (33.9 years old). Perceptions on the CFIO device cannot be generalized to all health care professionals working in the prehospital field or to other health care settings.

## Conclusion

Use of CFIO device is appreciated and well-perceived by paramedics during adult OHCA care. Paramedics reported that CFIO simplifies and expedites the resuscitation efforts by freeing a paramedic previously required to perform manual ventilation. The main reported disadvantages were the bending of the SGA device distal end and the loss of accurate capnography values. The impact of CFIO device on patient-important outcomes such as survival and survival with good neurological function needs to be investigated.

## Supplemental Material

sj-pdf-1-smo-10.1177_20503121211018105 – Supplemental material for Continuous flow insufflation of oxygen compared with manual ventilation during out-of-hospital cardiac arrest: A survey of the paramedics Click here for additional data file. Supplemental material, sj-pdf-1-smo-10.1177_20503121211018105 for Continuous flow insufflation of oxygen compared with manual ventilation during out-of-hospital cardiac arrest: A survey of the paramedics by Mathieu Groulx, Alexandra Nadeau, Marcel Émond, Jessica Harrisson, Pierre-Gilles Blanchard, Douglas Eramian and Eric Mercier in SAGE Open Medicine
