# Supplementary material for: Continuous flow insufflation of oxygen compared with manual ventilation during out-of-hospital cardiac arrest: A survey of the paramedics
Source: SAGE Open Med. 2021 Jun 30;9:20503121211018105. doi: 10.1177/20503121211018105 (PMC8252405; doi:10.1177/20503121211018105)
Supplement: sj-pdf-1-smo-10.1177_20503121211018105 – Supplemental material for Continuous flow insufflation of oxygen compared with manual ventilation during out-of-hospital cardiac arrest: A survey of the paramedics [file sj-pdf-1-smo-10.1177_20503121211018105.pdf]

B-card feedback survey

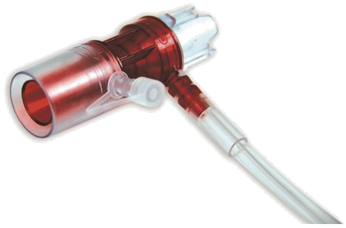

Q.1 How old are you?

____

Q.2 What is your gender?

	Female
	Male
	Other (please specify):

Q.3 How many years of experience as an EMT do you have (excluding years of education)?

____

Q.4 Have you ever performed a non-traumatic cardiac arrest resuscitation (RES-1 protocol) before the b-card was introduced?

	Yes
	No

Q.5 How many times have you performed a non-traumatic cardiac arrest resuscitation (RES-1 protocol) within the last 12 months?

____

Q.6 In how many interventions have you used the b-card since it's inception?

____

Q.7 Of all your interventions with the b-card, in how many cases was a supervisor present?

____

Q.8 Of all your interventions with the b-card, in how many cases were First Responders present?

____

Q.9 Rate how strongly you agree with this statement: I believe that I have received adequate training to use the b-card.

Totally agree	Partially agree	Neutral	Partially disagree	Totally disagree

The following questions are intended to capture your opinion on the new resuscitation protocol (RES-1) that introduced the use of the b-card, compared to the previous version of the protocol without the b-card.

Q.10 The use of the b-card improves **patient's** safety during the intervention compared to the standard ventilation strategy.

Totally agree	Partially agree	Neutral/Both methods are equivalent	Partially disagree	Totally disagree

Q.11 On site, using a b-card is safer for the **EMT** than using standard ventilation.

Totally agree	Partially agree	Neutral/Both methods are equivalent	Partially disagree	Totally disagree

Q.12 During transportation, using a b-card is safer for the **EMT** than using standard ventilation.

Totally agree	Partially agree	Neutral/Both methods are equivalent	Partially disagree	Totally disagree

Q.13 During victim's evacuation, using a b-card is safer for the **EMT** than using standard ventilation.

Totally agree	Partially agree	Neutral/Both methods are equivalent	Partially disagree	Totally disagree

Q.14 EMS procedure is faster with the b-card compared to standard ventilation.

Totally agree	Partially agree	Neutral/Both methods are equivalent	Partially disagree	Totally disagree

Q.15 EMS procedure is easier to perform with the b-card compared to standard ventilation strategy.

Totally agree	Partially agree	Neutral/Both methods are equivalent	Partially disagree	Totally disagree

Q.16 I feel less physical fatigue after an operation using the b-card instead of standard ventilation strategy.

Totally agree	Partially agree	Neutral/Both methods are equivalent	Partially disagree	Totally disagree

Q.17 I feel less mental fatigue after an operation using the b-card instead of standard ventilation strategy.

Totally agree	Partially agree	Neutral/Both methods are equivalent	Partially disagree	Totally disagree

Q.18 Overall, I prefer using the b-card for non-traumatic cardiac arrest operations compared to standard ventilation strategy.

Totally agree	Partially agree	Neutral/Both methods are equivalent	Partially disagree	Totally disagree

Q.19 Did you add 20 cc of air into proximal cuff when using the b-card?

	Yes (Specify the number of events):
	No

Your comments on the b-card would be greatly appreciated

Q.20 What problem(s) occurred while using the b-card?

____

____

____

Q.21 Which element(s) of the b-card facilitate patient’s care or cardiac arrest management?

____

____

____

Q.22 Any additional comments?

____

____

____

Thank you for your input!
